# Assessment of food hygiene and safety knowledge, attitude, and practices of fruit and vegetable vendors in the Ho central market, Ghana

**DOI:** 10.1016/j.heliyon.2023.e19579

**Published:** 2023-08-29

**Authors:** Maxwell Kwame Boakye, Janet Mawunyo Tornyi, Innocent Dzubey, Paulina Adzoyi, John Coker Ayimah, Dorothy Serwaa Boakye, Edward Debrah Wiafe

**Affiliations:** a Department of Environmental Science, Ho Technical University, Ho, Ghana; b School of Hygiene, Ho, Ghana; c Department of Hospitality and Tourism Management, Ho Technical University, Ho, Ghana; d Department of Mathematics and Statistics, Ho Technical University, Ho, Ghana; e Department of Health Administration and Education, University of Education, Winneba, Ghana; f School of Natural and Environmental Sciences, University of Environment and Sustainable Development, Somanya, Ghana

**Keywords:** Storage facilities, Health education, Public awareness, Market amenities, Insecticides

## Abstract

Local markets are primarily the center for trade and distribution of fruits and vegetables in Ghana. Fruit and vegetable vendors are responsible for keeping food hygienic and safe from contamination. However, little is known about fruit and vegetable vendors' knowledge, attitudes, and practices (KAP) toward food hygiene and safety in local markets. This study aimed to assess the KAP of fruit and vegetable vendors in Ho City. Data on KAP was collected from 113 fruit and vegetable vendors in the Ho Central Market using a structured questionnaire. The data was analyzed using a two-way multivariate analysis of variance (MANOVA) and one-way analysis of variance (ANOVA) to examine the association of demographic variables with knowledge, attitude, and practice of food hygiene and safety. A correlation analysis was conducted to determine the intercorrelation among the KAP variables. The results suggest significant differences for vendors with medical examination certificates on knowledge (Wilks = 0.60, *F* = 2.82, *p*˂0.00), attitude (Wilks = 0.71, *F* = 2.10, *p*˂0.01), and practice (Wilks = 0.59, *F* = 1.79, *p*˂0.01). A significant correlation was found between the three domains, but the influence of knowledge and attitude on practice was weak. Fruit and vegetable vendors' knowledge and attitudes toward food hygiene and safety were supportive and favorable. However, some practices were not supportive and encouraging toward food hygiene and safety. The lack of basic amenities in the market influenced the practice of vendors. Improved environmental sanitation conditions at local markets are vital to the practice of food hygiene and safety to prevent foodborne diseases.

## 1 Introduction

Fruits and vegetables are rich sources of vitamins and minerals that help in promoting good health [[1], [2], [3]]. Local markets serve as the primary centers for the distribution of fruits and vegetables [[4], [5], [6], [7], [8], [9], [10]]. Vendors handling practices of fruit and vegetables have been identified as a potential source of contamination [8,11,12]. Handling practices by vendors, including method of display, mode of transport, storage, and sprinkling fresh vegetables with non-potable water, are significant sources of contamination [[5], [6], [7], [8], [9]]. Thus, fruit and vegetable consumption can be a source of potential pathogenic infection leading to foodborne diseases [12,13] from postharvest handling practices by vendors.

Local fruit and vegetable vendors are responsible for keeping food hygienic and safe from contamination. The ability of the vendors to ensure food hygiene and safety is shaped by their knowledge, attitudes, and practices (KAP) [14]. However, little information is available regarding the KAP of fruit and vegetable vendors in local markets towards hygiene and safety in Ghana. Available literature on the KAP of fruit and vegetable vendors in Ghana [[5], [6], [7], [8], [9],^15^] did not comprehensively assessed the knowledge, attitude, and practice of vendors concurrently, as done in the case for KAP research on ready-to-eat street and institutional foods handlers [[16], [17], [18]]. The previous studies on fruit and vegetable vendors' food hygiene and safety focused on one or at most two constructs of KAP. The association between knowledge, attitude, and practice of food hygiene and safety among Ghanaian fruit and vegetable vendors has yet to be discovered. The purpose of this study is to assess whether knowledge promotes a positive attitude, which, in turn, shapes the practice of food hygiene and safety of fruit and vegetable vendors in Ghana.

Local markets in Ho city are primarily the nerve center for the trade and distribution of fruits and vegetables but are identified with unhygienic and unsafe handling practices among vendors [19,20]. Vendors' fruit and vegetable postharvest handling activities, including mode of transporting, packaging, and display, contributed significantly to contamination at the various local markets in Ho city [20]. However, studies are yet to be undertaken to determine the KAP of the fruit and vegetable vendors in Ho city. The city is the administrative capital of the Volta Region and serves as the center of commerce. Also, previous studies on the KAP of fruit and vegetable vendors in Ghana were undertaken in traditionally established larger markets in major cities [[5], [6], [7], [8], [9]], creating an information deficit for smaller cities not known for their large markets. Therefore, understanding vendor KAP is crucial in designing interventions to improve food hygiene and safety, which could have an immense public health benefit. The study aimed to assess food hygiene and safety KAP among fruit and vegetable vendors in the Ho Central Market, given their crucial roles in reducing foodborne illnesses. The objectives of the study were to determine whether fruit and vegetable vendors (a) have satisfactory knowledge of food hygiene and safety, (b) have satisfactory attitudes and practices toward food hygiene and safety, and (c) knowledge influences attitude and whether that attitude translates into practice. The findings from this study can help improve existing protocols and be used to develop food hygiene and safety intervention strategies.

## 2 Materials and methods

### 2.1 Study area

The study was conducted in the Ho Central Market (HCM), located in the Ho Municipality of the Volta Region. The municipality, which covers a total land area of 587 km^2^ and has a human population of 180,420, lies between latitudes 6°20″N and 6° 55″N and longitudes 0°12′E and 0° 53′E [21]. The HCM, popularly known as ‘Ho Asigame’ (Ho big market), is one of the biggest trading centers in the region and a central hub for commercial activities in the municipality [22,23]. The HCM is the leading center for distributing fruits and vegetables as vendors receive supplies from several rural farmers and a chain of vastly interconnected market intermediaries [19,20]. Previous studies have identified improper handling of vegetables by vendors in the HCM [19,20]. However, no previous studies have assessed the KAP of fruit and vegetable vendors in the HCM, which has necessitated this study.

### 2.2 Instrument for data collection

The questions were adapted after reviewing the relevant literature on food vendors' knowledge, attitudes, and practices (KAP) toward food hygiene and safety to identify the relevant items and scales in the existing questions. The questionnaire was adapted from these related studies [8,9,17,18,[24], [25], [26], [27]]. The content validity of the questionnaire was ascertained by five experts in the related field from the School of Hygiene, Ho, and the Department of Food Science at Ho Technical University, who assisted with selecting the most appropriate questions from the adapted studies. Minor textual adjustments were made to suit the focus of this study without any major changes to the study instruments adapted. Accordingly, 21, 18, and 14 indicators were selected for knowledge, attitude, and practice. The first part of the questionnaire dealt with demographic characteristics such as gender, age group, last level of formal education, experience in fruit and vegetable vending, and employment status. The second part of the questionnaire dealt with the type of fruit and vegetable sold, mode of preservation of products, source of water, the place for storage of products, training on food hygiene and safety, and medical examination certificate. The third section of the questions dealt with fruit and vegetable vendors' knowledge, attitudes, and practices (KAP). Participants were asked to rate their degree of agreement with the indicators of knowledge and attitude on a five-point Likert scale varying from "Strongly disagree" (1) to "Strongly agree" (5). Regarding knowledge and attitude indicators, strongly agree and agree responses were regarded as satisfactory or favorable of food hygiene and safety. The frequency of practice of food hygiene and safety indicators was rated on a five-point Likert scale varying from "Never" (1) to "Always" (5). Always and often answers were considered supportive or favorable toward food hygiene and safety practice.

### 2.3 Pilot study

The adapted questions were pretested on fruit and vegetable sellers in the Ho Civic Market near the HCM. Most vendors in the civic market display their goods in the central market during market days and have similar KAP toward food hygiene and safety. A total of 23 respondents were selected through convenient sampling for the pilot study. The Cronbach alpha test was used to analyze the reliability of the piloted questionnaire, and a score within the acceptable range limit of (>0.7) was observed for knowledge, attitude, and practice constructs. The Cronbach alpha scores demonstrated the presence of adequate construct reliability in the questionnaire's accuracy and suitability for the study. The pilot sample was excluded from the study's main sample.

### 2.4 Determination of sample size

A preliminary survey was conducted on 30 conveniently sampled individuals in the study market. It was found that 28 individuals (*p* = 0.93) knew about fruit and vegetable vending in the HCM. This information was used to calculate the sample size according to the formula of Dagnelie [28] in equation (1):

(1) n=U1−∝22×p(1−p)d2

where U1−∝2 is the value of the Normal random variable corresponding to a probability value of 1−∝2. For a probability value of 0.975 (∝ = 0.05), U1−∝2 ≈ 1.96; d is the margin error of the estimation of any parameter to be computed, which was fixed at 5% (0.05). Under these assumptions, the sample size to use was established to be 100 people but was increased to 113 due to the cooperation of the study respondents.

### 2.5 Sampling procedure and data collection

A purposeful sampling approach, which involves selecting participants who could provide information pertinent to the study, and a snowball sampling approach, which uses a targeted population to recommend other members of that population [29] were used in this study. This approach ensured that the participants selected were representatives of the fruit and vegetable trade in the HCM and could provide information pertinent to this study. The sampling approaches were complimented by exhaustive walking along all the available lanes, corners, and streets surrounding the market where fruit and vegetables are known to be sold to ensure coverage of the obscured vendors.

Market days where buyers and sellers converge on a given location to trade on a periodic basis [30] were chosen for the data collection. The HCM is a center of attraction on market days which, unlike the regular days, displays large quantities of foodstuff, including fruits and vegetables, at very affordable prices [22]. Data were collected from respondents through face-to-face interviews using a structured questionnaire on market days in November and December 2022. Face-to-face administering of the questionnaire was employed due to the low literacy levels of most fruit and vegetable market vendors in Ghana [[5], [6], [7], [8], [9],^15^]. The data collectors explained the content of the questionnaire in the Ewe local language, widely spoken in the municipality and adopted by many others as the lingua franca to the respondents who could not read and understand the English language. All the data collectors were fluent in English and Ewe languages. The data collectors practiced reading the questions aloud to the satisfaction of the experts from the School of Hygiene, Ho, and the Department of Food Science at Ho Technical University in English and Ewe languages for the pilot and main study. Direct observations were used to verify information provided by the stakeholders on hygienic practices.

### 2.6 Data analysis

Descriptive analysis was performed to determine count and percentages for demographic factors, mode of preservation of products, source of water in the market for washing fruit and vegetables, the place for storage of products, training on food hygiene and safety, and medical examination certificate. A word cloud visual representation of word frequency was used to represent the fruits and vegetables sold in the HCM using WordIt Out. The sizes of the words are proportional to the frequency of mentions by the vendor.

A multivariate analysis of variance (MANOVA) was used to determine the extent of the association of dependent variables (knowledge, attitude, and practice) with the independent variables (educational level, experience, training, and medical certification). A MANOVA was used because there were more than one dependent variable and more than two independent variables. The composite scores of the three dependent variables (under the assumption of normality) were determined and used in the MANOVA. A significant MANOVA test suggest an analysis of variance (ANOVA) be conducted to determine where differences exist. The inter-correlation between respondents' knowledge, attitudes, and practices was determined. All statistical analyses were performed using the SPSS version 22 and the R statistical software at a 5% significance level.

## 3 Results

**Fig. 1:**
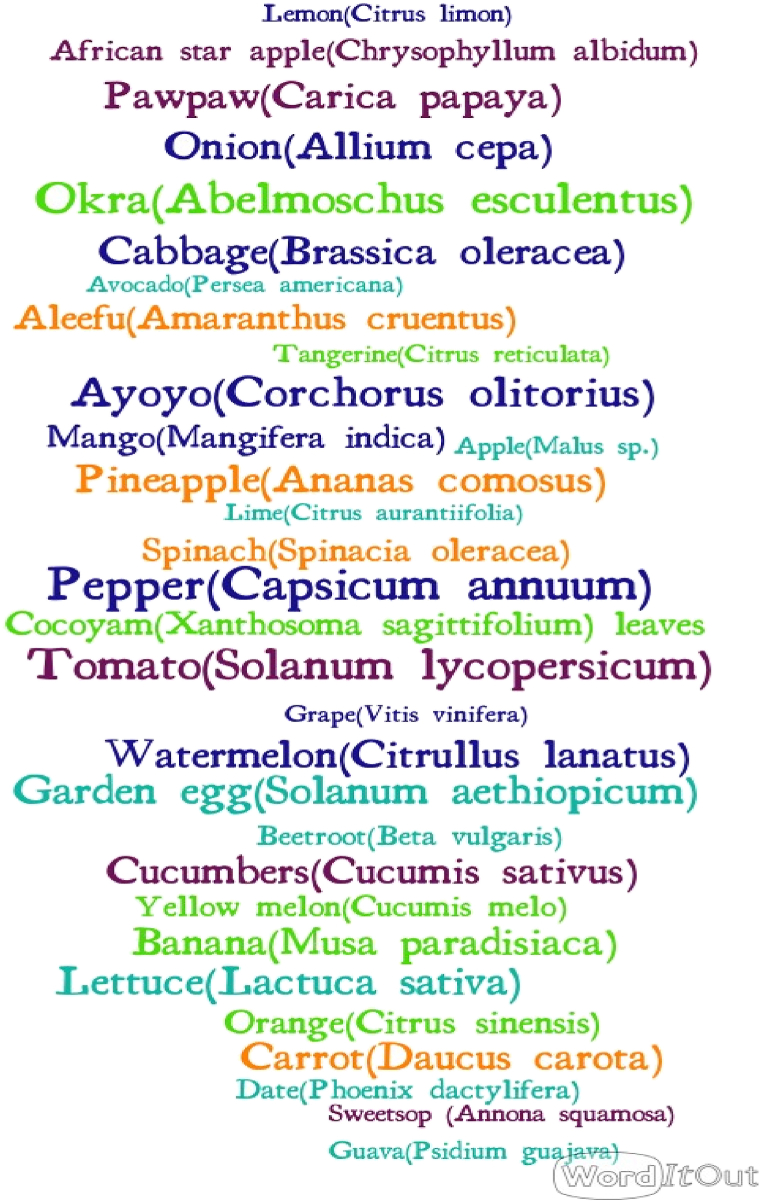
Word cloud of the fruits and vegetables mentioned by traders as being sold in the Ho Central Market. Maximum mentions are 91 for pepper.

Table 1 shows that the majority of the fruit and vegetable vendors in the HCM were females (n = 106; 93.8%), had completed JHS (n = 57; 50.4%), and had a work experience of 3–5 years. The age group of the respondents indicates that the majority were between the ages of 21–50 years, and the respondents' common form of employment was permanent (n = 63; 55.8) (Table 1). Keeping fruits and vegetables in a cool dry place (n = 106; 93.8) was vendors' most common method for perseveration. The majority of vendors stored their products at home at the end of the day's trade (n = 87; 77.0%), followed by private storehouses outside the market (n = 17; 15.0), private storehouses inside the market (n = 8; 7.1%), under personal stand in the market (n = 1; 0.9). Most respondents have yet to undergo medical screening, do not have a medical certificate (n = 69; 61.1%), and have not participated in any food hygiene and safety training (n = 104; 92.0%). The highest number of mentions for fruit and vegetable traded by vendors was recorded for pepper (*Capsicum annuum;* n = 91) followed by okra (*Abelmoschus esculentus;* n = 80), tomato (*Solanum lycopersicum;* n = 79), ayoyo (*Corchorus olitorius;* n = 75), garden egg (*Solanum aethiopicum;* n = 70) while the least number of mentions was for avocado (*Persea americana*) (n = 8) (Fig. 1).

**Table 1:** Socio-demographic characteristics of vendors.

*Gender of vendor*	Count	%
Male	7	6.2
Female	106	93.8
*Age groupings of vendors*		
18–20	11	9.7
21–30	27	23.9
31–40	33	29.2
41–50	28	24.8
51–60	12	10.6
>60	2	1.8
*Highest educational level of vendors*		
Below Primary	6	5.30
Primary	16	14.2
JHS	57	50.4
MSLC	13	11.5
SHS/TEC	19	16.8
Tertiary	2	1.8
*Work experience of vendors*		
1–2	20	17.7
3–5	43	38.1
6–10	25	22.1
11–15	11	9.7
16–20	7	6.2
Above 20	7	6.2
*Employment status of vendors*		
Permanent	63	55.8
Temporal	32	28.3
Casual	11	9.7
Seasonal	7	6.2
*Type of uncooked foodstuff sold*		
Fruits	39	34.5
Vegetables	64	56.6
Both	10	8.8
*Mode of fruit and vegetable preservation*		
Keeping in a cool, dry place	106	93.8
Refrigeration	7	6.2
*Do you wash your products before sale*		
Yes	46	40.7
No	67	59.3
*Where do you store the products at the end of the day?*		
At home	87	77.0
Private storehouse outside the market	17	15.0
Private storehouse inside the market	8	7.1
Under private stand in the market	1	0.9
*Main sources of water for washing fruits and vegetables*		
Public stand pipe	60	53.1
Stored water in a gallon	29	25.7
Sachet water	22	19.5
Borehole	2	1.6
*Have you participated in any food safety training?*		
Yes	9	8.0
No	104	92.0
Do you have a health certificate?		
Yes	44	38.9
No	69	61.1

Note: JHS = junior high school; SHS = senior high school/technical; MSLC = middle school leaving certificate.

Table 2 presents the mean ratings for the 21 indicators used to assess respondents' food hygiene and safety knowledge. The maximum mean scores of 4.76 and a minimum of 4.59 indicate that the vendors strongly agree or agree with the variables and, therefore, are satisfactory for food hygiene and safety. Washing hands at work to reduce the risk of external contamination, and insects, such as cockroaches and flies, might transmit foodborne pathogens had the highest mean (4.76; each), followed by foodborne disease affects all, including infants, teenagers, pregnant women, and the elderly and *Salmonella* is among the foodborne pathogens (4.74; each). A clean working environment is essential for preventing contamination, and raw fruits and vegetables are stored away from, and not next to, or below, raw meat, poultry, and seafood (4.72; each). The knowledge means for foodborne disease occurrence was higher, followed by personal hygiene, contamination, temperature, and quality control.

**Table 2:** Knowledge base of fruit and vegetable vendors.

Knowledge indicators	Mean	SD
X1 = Washing hands at work reduces the risk of external contamination	4.76	.62
X2 = While coughing or sneezing, it is necessary to cover your mouth	4.66	.55
X3 = Handlers with wounds, abrasions, and cuts should not be handling fruits and vegetables	4.67	.53
X4 = Good hygiene practices can help to avoid foodborne diseases	4.69	.50
X5 = A clean working environment is essential for the prevention of contamination	4.72	.51
X6 = Handlers with diseases such as diarrhea, flu, and sore throat risk fruit and vegetable contamination	4.65	.55
X7 = Wearing gloves while handling fruits and vegetables reduces the risk of contamination	4.68	.54
X8 = The use of a cap, masks, and adequate clothing can reduce the risk of contamination of fruits and vegetables	4.65	.51
X9 = Wearing adornments like earrings, rings, and watches can cause contamination	4.71	.49
X10 = Eating and drinking at the workplace increase the risk of food contamination	4.65	.55
X11 = Raw fruits and vegetables must be kept under a temperature of 40Â°F (4Â°C) in a refrigerator	4.63	.52
X12 = Raw fruits and vegetables are stored away from, and not next to or below, raw meat, poultry, and seafood	4.72	.45
X13 = Contaminated fruits and vegetables vary in color, texture, odor, or taste	4.66	.59
X14 = Unhygienic handling of fruits and vegetables can cause foodborne diseases	4.68	.52
X15 = Foodborne disease affects all, including infants, teenagers, pregnant women, and the elderly	4.74	.58
X16 = Cross-contamination is when microorganisms are transferred from the handler hands or utensils to another person	4.59	.62
X17 = Salmonella is among the foodborne pathogens	4.74	.50
X18 = Hepatitis A virus is among the foodborne pathogens	4.69	.57
X19 = Insects, such as cockroaches and flies, might transmit foodborne pathogens	4.76	.43
X20 = Healthy fruits and vegetable handlers may still carry foodborne pathogens	4.71	.48
X21 = Proper disposal of fruits and vegetable wastage reduces the risk of microbial spread and contamination	4.67	.60

The attitude indicators assessed were supportive or favorable for food hygiene and safety, with most respondents strongly agreeing or agreeing with the variables (Table 3). The variable with the highest mean was cleaning the work area before starting work (4.81). Handlers who have cuts, scratches, or injuries to their hands were forbidden from touching or handling fruit and vegetable, proper fruit and vegetable preservation were critical for food safety, and keeping shop, knives, and cutting boards clean reduces the risk of foodborne diseases had the second highest mean (4.78; each).

**Table 3:** Attitude of fruit and vegetable vendors.

Attitude indicators	Mean	SD
Y1=The work area must be cleaned before starting work	4.81	.73
Y2 = Maintaining a high degree of personal hygiene at the workplace is vital	4.59	.66
Y3=Handlers who have cuts, scratches, or injuries to their hands are forbidden from touching or handling fruit and vegetable	4.78	.65
Y4=Wearing gloves is a necessary precaution to minimize the chance of contamination	4.63	.64
Y5=Wearing caps and adequate clothing is a necessary precaution to minimize the chance of contamination	4.69	.63
Y6=Proper fruit and vegetable preservation is critical for food safety	4.78	.53
Y7=Spoiled or contaminated fruit and vegetable should be discarded immediately	4.73	.46
Y8=Safe fruit and vegetable handling to avoid contamination is a crucial aspect of my job responsibility	4.72	.53
Y9=Washing hands before handling fruits and vegetables is essential to reduce contamination	4.72	.51
Y10=I will change my fruit and vegetable handling behaviors when I know they are incorrect	4.73	.50
Y11=I will participate in a training programme to improve fruit and vegetable safety and hygiene practices	4.73	.48
Y12=Keeping shop, knives, and cutting boards clean reduces the risk of foodborne diseases	4.78	.44
Y13=Flies, insects, or rodents can cause fruit and vegetable bacterial contamination	4.76	.45
Y14=Using insecticides to manage insect infestation is very harmful to human health	4.75	.47
Y15=Food handlers can be a source of foodborne outbreaks	4.65	.55
Y16=It is necessary to check the temperature of refrigerators/freezers periodically to reduce the risk of food contamination	4.71	.59
Y17=Raw fruits and vegetables are not free from pathogenic microorganisms	4.73	.46
Y18=Raw, unwashed vegetables pose a high risk of foodborne diseases	4.73	.66

Table 4 indicates the mean scores for practice indicators of food hygiene and safety. The most frequent practice was cleaning the working area before and after handing out fruits and vegetables, with a mean score of 4.70. The second most common practice for food hygiene and safety was wearing an apron while working, followed by ensuring that the fruits and vegetables sold were not on bare ground (4.54) and ensuring that fruits and vegetables were not exposed to excessive temperature or sunshine (4.40). The use of any insecticides during the fruits and vegetable handling process had the least mean score (3.27), which is not supportive or favorable for food hygiene and safety as most respondents sometimes practice this behavior. The mean scores for using protective gloves while handling fruits and vegetables (3.80) and washing hands properly before or after using gloves (3.74) were also not supportive or favorable for food hygiene and safety practices. The average mean score for food hygiene and safety practices was lower than the knowledge and attitude scores.

**Table 4:** Safety practices undertaken by vendors.

Practices	Mean	SD
Z1 = Do you wash your hands properly after handling money	4.31	1.05
Z2 = Do you cover your mouth with tissue paper while coughing or sneezing	4.37	.72
Z3 = Do you wash your hands before and after rubbing your nose or scratching your body	4.27	.87
Z4 = Do you use protective gloves while handling fruits and vegetables	3.80	1.51
Z5 = Do you wash your hands properly before or after using gloves	3.74	1.59
Z6 = Do you wear an apron while working	4.65	.75
Z7 = Do you handle fruits and vegetables with a wounded hand that is not entirely covered	4.14	1.16
Z8 = Do you properly clean the working area before and after handing out fruits and vegetables	4.70	.60
Z9 = Do you wash your hands after handling spoiled or contaminated fruits and vegetables	4.41	.87
Z10 = Do you bother discarding spoiled and contaminated fruits and vegetables^a^	4.35	.82
Z11 = Do you use any insecticides during the fruits and vegetable handling process^a^	3.27	1.81
Z12 = Do you wash fruits and vegetables with clean water before selling them	4.12	1.27
Z13 = Do you ensure that the fruits and vegetables sold are not on bare ground	4.54	.85
Z14 = Do you ensure that fruits and vegetables are not exposed to excessive temperature or sunshine	4.40	.87

a Reverse coded.

The high Cronbach's alpha values for knowledge (0.88), attitude (0.91), and practice (0.82) variables suggest the strong coherence and consistency among the indicators of the three variables. The multivariate analysis of variance (MANOVA) test shown in Table 5 suggests significant differences in knowledge, attitude, and practice for participants who have undergone medical screening and received health certificates (*p* = 0.00, 0.01and 0.00), respectively. There appears to be some significant difference (*p=*0.03) between those who participated in food hygiene and safety training and those who did not. However, a further ANOVA revealed no significant difference (*p* = 0.22). Regarding the relationship among the three variables, the correlation matrix showed that 31.6% of the time, knowledge acquired by vendors informs the attitude they put up, and 20% of the time, their knowledge influences what they practice (Table 6). However, attitude influences practice only 9% of the time.

**Table 5:** Socio-demographic association with knowledge, attitude and practice (MANOVA and ANOVA).

Variable	Demographic characteristics	Cronbach's alpha (α	MANOVA			ANOVA			
			Wilks	F	Sig.	Grand mean	Group mean	Differing groups	Sig.
Knowledge	Education	0.88	0.82	0.90	0.59	98.45	No significant difference between groups		
	Experience		0.78	1.16	0.31		No significant difference between groups		
	Employment		0.80	1.06	0.41		No significant difference between groups		
	Training		0.76	1.32	0.18		No significant difference between groups		
	Certification		0.60	2.82	0.00^a^		1 = 101.6 2 = 96.43	1 and 2	0.00^a^
Attitude	Education	0.91	0.90	0.56	0.92	85.04	No significant difference between groups		
	Experience		0.84	0.96	0.51		No significant difference between groups		
	Employment		0.83	1.00	0.46		No significant difference between groups		
	Training		0.76	1.63	0.07		No significant difference between groups		
	Certification		0.71	2.10	0.01^a^		1 = 87.36 2 = 83.55	1 and 2	0.00^a^
Practice	Education	0.82	0.94	0.44	0.96	59.08	No significant difference between groups		
	Experience		0.80	1.66	0.08		No significant difference between groups		
	Employment		0.81	1.60	0.09		No significant difference between groups		
	Training		0.77	1.97	0.03^a^		1 = 62.44 2 = 58.79	1 and 2	0.22
	Certification		0.59	1.79	0.00^a^		1 = 63.09 2 = 56.52	1 and 2	0.00^a^

a Significant at p <0.05.

**Table 6:** Correlation matrix between knowledge, attitude and practice.

Variable	Correlation (Multiple R-squared)		
	Knowledge	Attitude	Practice
Knowledge	1		
Attitude	0.56(31.6%)^a^	1	
Practice	0.45(20.0%)^a^	0.30 (9%)^a^	1

a Correlation significant at p < 0.05.

## 4 Discussion

The unavailability of storage structures for fruit and vegetables in this study has been identified in the previous research in the Ho Central Market [19,20] and other local markets in Ghana [[5], [6], [7],^10^]. The lack of storage structures resulted in many vendors storing their commodities at home in this study. Frequent transportation of fruit and vegetable has implications on hygiene and safety. Improper handling, temperature control, and loading practices are some of the critical food hygiene and safety hazards associated with transportation [5,31]. Kumah [20] identified transportation as a major source of contamination of vegetables in the Ho Municipality as the means of transport are not adequately cleaned before loading. The lack of cleaning of transport as a possible source of contamination of fruits and vegetables has been mentioned in other local markets [32]. Vendors' frequent transportation between the home and the market exposes fruits and vegetables to high chances of physical, chemical, and microbial contamination. Inadequate storage facilities may have facilitated the spoilage of vegetables, which led to great concern in discarding spoiled and contaminated fruits in this study. Kushitor et al. [5] found the spoilage rate of vegetables to be a major concern among vendors in Kumasi due to the lack of appropriate means and space to store the vegetables.

Fruit and vegetable vendors' high knowledge of hygiene in this study was consistent with other studies in Ghana [8,9,15]. Public awareness campaigns on food hygiene and safety are standard practices in local markets in Ghana. Food hygiene and safety awareness programs were increased during the COVID-19 pandemic to minimize the impact of foodborne illnesses [[33], [34], [35]] and in local markets in Ghana [[36], [37], [38]]. The high knowledge of foodborne illnesses transmission in this study can be attributed to the COVID-19 pandemic awareness creation. Vendors presenting themselves for medical screening offered an opportunity for health workers to provide health education, which resulted in significantly higher KAP of vendors with medical certificates. The higher KAP of vendors with medical examination certificates is corroborated by Mohammed et al. [8] study that found a high food hygiene and safety knowledge for fruit and vegetable vendors with medical certificates.

Cleaning the workplace before starting work is a common hygienic practice among food vendors in Ghana [8,9,39]. The high mean for KAP towards cleaning the working area was compatible with the observation made in other studies [8,9]. Ensuring that fruits and vegetables sold are not on the bare ground and exposed to excessive temperature or sunshine was consistent with [9]. The low adherence to gloves usage in handling fruits and vegetables was accordant with observations from other studies in Ghana [9,20]. The high mean for handling fruit and vegetables with a wounded hand that is not adequately covered and the nonadherence to glove use which could have covered wounds and abrasions, is a serious public health concern. Vendors are required not to handle food as a wound can be a potential source of food contamination and transmission routes of several types of communicable diseases.

In most local markets in Ghana, basic facilities and services such as drainage, regular rubbish collection, and clean toilets are either poor or non-existent [5,8,9,40]. Flies and other pathogen-carrying insects hover over edibles displayed items in local markets as a result of the unsanitary conditions [8,9,32]. Exposure to houseflies and pathogenic insects may have influenced the favorable response toward using insecticides during fruit and vegetable handling. Overå et al. [41] identified the spraying of insecticides to control houseflies and insects by fishmongers in local markets in Ghana. Indeed, the local market settings of low environmental and sanitary standards contribute to the difficulty of maintaining and sustaining food hygiene and safety practices. Previous studies in Ghana have also found limitations with facilities in local markets as a hindrance to the practice of food hygiene and safety by fruit and vegetable vendors [[5], [6], [7],^10^].

Education and training are major strategies for imparting KAP regarding food hygiene and safety [17,35,44]. However, this study did not observe any impact of education and training on KAP. The lack of education on KAP can be attributed to the low level of formal education of the respondents in this study, which did not offer them the opportunity to learn about food hygiene and safety. A similar observation was made by Addo-Tham et al. [45] in Ghana, whereby the level of education did not have an impact on the KAP of food hygiene and safety of street food vendors. The lack of influence of training on KAP is supported by previous studies [39,46] that assert that training vendors in safe food handling are insufficient in achieving compliance. The everyday context seems to influence food handling hygiene and safety significantly [39,46].

This study conformed with the KAP model, which suggests that knowledge can promote a positive attitude, and that attitude, in turn, shapes the practice of food hygiene and safety [25,42]. The fruit and vegetable vendors with sufficient knowledge showed a positive attitude and practices toward food hygiene and safety. However, the influence of knowledge and attitude is not that strong, indicating that vendors may not necessarily practice food hygiene and safety procedure during food handling even though their responses indicate a high level of knowledge and attitude towards hygiene and safety. According to da Cunha et al. [42], various factors influence the KAP model by limiting the translation of knowledge into appropriate practices. Clayton et al. [43] identified a lack of resources as a barrier that prevents food handlers from implementing hygiene practices. In the context Ghanaian local market, the lack of basic facilities and services may be the primary determinant hindering the practice of food hygiene and safety. Improved infrastructure and the provision of basic amenities to facilitate hygienic practices in the local market are crucial to promoting food hygiene and safety. The limitations associated with the KAP model [42,47] may have influenced the associations between the constructs. Despite the limits of the KAP model, it remains the commonly applied method to food safety-related investigations [42,47].

## 5 Conclusion

This study examined the KAP of fruit and vegetable vendors considering their pivotal role in the food supply chain and its impact on food hygiene and safety. The study's findings revealed that vendors had satisfactory knowledge and attitude toward food hygiene and safety but were not reflected in their practices. The lack of basic amenities in the market generally limited the practice food hygiene and safety by the vendors. The local government needs to improve the environmental conditions to support the practice of food hygiene and safety in local markets to prevent foodborne diseases. The application of insecticides and handling fruits and vegetables with a wounded hand that is not entirely covered were practices that have public health implications. Market vendors need to be sensitized about the health effects of using insecticides on food.

The findings of the study had some limitations. The findings are based on self-reported practices by fruit and vegetable vendors in one market in the Volta Region. Hence, the results cannot be generalized to all the regional or country markets. Therefore, it will be helpful to extend the study to other markets in the region, particularly in smaller markets with infrastructure deficits and limited monitoring from the health inspectors, to gain insights into KAP of food hygiene and safety in future studies. Also, the cross-sectional design of this study does not determine causality but an association among the variables used at a single point in time. Future researchers may need to adopt a longitudinal approach to assess the pattern of change in food hygiene and safety knowledge, attitude, and practice changes over time. The survey was conducted on market days, and vendors who did not sell could have been excluded. Future studies should consider daily market visits to ascertain the pattern of change, if any. Despite these limitations, the study has the strengths of concurrently assessing the knowledge, attitude, and practice of food hygiene and safety among fruit and vegetable market vendors, which was lacking.

## Funding statement

This research did not receive any specific grant from funding agencies in the public, commercial, or not-for-profit sectors.

## Additional information

No additional information is available for this paper.

## Consent to participate

Informed consent was obtained from all individual participants included in the study.

## Ethics statement

This study was approved by the ethics committee of Ho Technical University (HTU/2022/10/010). An oral informed consent was obtained from all participants interviewed. Traditional protocol, such as approval of the study by the market queen of HCM, was obtained before the face-to-face questionnaire interviews were administered.

## Author contribution statement

Maxwell Kwame Boakye: Conceived and designed the experiments; Performed the experiments; Analyzed and interpreted the data; Contributed reagents, materials, analysis tools or data.

Janet Mawunyo Tornyi, Innocent Dzubey: Conceived and designed the experiments; Performed the experiments; Contributed reagents, materials, analysis tools or data; Wrote the paper.

Paulina Adzoyi, Dorothy Serwaa Boaakye, Edward Debrah Wiafe: Conceived and designed the experiments; Contributed reagents, materials, analysis tools or data; Wrote the paper.

John Coker Ayimah: Conceived and designed the experiments; Analyzed and interpreted the data; Wrote the paper.

## Data availability statement

Data included in article/supplementary material/referenced in article.

## Declaration of competing interest

The authors declare that they have no known competing financial interests or personal relationships that could have appeared to influence the work reported in this paper.
